# Functionalized copolyimide membranes for the separation of gaseous and liquid mixtures

**DOI:** 10.3762/bjoc.6.86

**Published:** 2010-08-12

**Authors:** Nadine Schmeling, Roman Konietzny, Daniel Sieffert, Patrick Rölling, Claudia Staudt

**Affiliations:** 1 Institute for Organic and Macromolecular Chemistry, Heinrich-Heine University of Duesseldorf, Universitaetsstr. 1, 40225 Duesseldorf, Germany

**Keywords:** aromatics/aliphatics, copolyimides, cross-linking, gas separation, membranes, natural gas treatment, olefins/paraffins, pervaporation

## Abstract

Functionalized copolyimides continue to attract much attention as membrane materials because they can fulfill the demands for industrial applications. Thus not only good separation characteristics but also high temperature stability and chemical resistance are required. Furthermore, it is very important that membrane materials are resistant to plasticization since it has been shown that this phenomenon leads to a significant increase in permeability with a dramatic loss in selectivity. Plasticization effects occur with most polymer membranes at high CO_2_ concentrations and pressures, respectively. Plasticization effects are also observed with higher hydrocarbons such as propylene, propane, aromatics or sulfur containing aromatics. Unfortunately, these components are present in mixtures of high commercial relevance and can be separated economically by single membrane units or hybrid processes where conventional separation units are combined with membrane-based processes. In this paper the advantages of carboxy group containing 6FDA (4,4′-hexafluoroisopropylidene diphthalic anhydride) -copolyimides are discussed based on the experimental results for non cross-linked, ionically and covalently cross-linked membrane materials with respect to the separation of olefins/paraffins, e.g. propylene/propane, aromatic/aliphatic separation e.g. benzene/cyclohexane as well as high pressure gas separations, e.g. CO_2_/CH_4_ mixtures. In addition, opportunities for implementing the membrane units in conventional separation processes are discussed.

## Introduction

Over 50% of energy costs in the chemical industry are used for the separation of gaseous or liquid mixtures [1]. Separations in petrochemical processes, e.g. low temperature distillation of olefin/paraffin mixtures or extractive distillation for the production of benzene as well as the separation of isomeric xylenes by low temperature fractional crystallization are highly energy intensive. If these separation processes could be improved, the costs of basic chemicals as ethylene, propylene and benzene could be drastically reduced.

Membrane devices offer new opportunities for the separation of gaseous or liquid mixtures. Compared to conventional distillation units, membrane devices are much smaller and processes can be conducted at lower temperatures. In certain cases, the combination of distillation and membrane units, so called hybrid processes, have been established. For the separation of olefin/paraffin mixtures it has been estimated that membrane based hybrid processes could save approximately 40–50% of the production costs [2]. The worldwide membrane market currently has a steady growth of approximately 10–15% each year [3]. Membrane based separations of gaseous mixtures have been well established for natural gas treatments (removal of carbon dioxide), for hydrogen removal (e.g. in cracking processes) for oxygen enrichment from air (medical devices) and for nitrogen enrichment from air (used as an inert atmosphere for oxygen sensitive compounds). Other areas with fast growing market relevance are vapor recovery systems [4], monomer recovery units, e.g. ethylene/nitrogen or propylene/nitrogen [5–6], the dehydration of organic solvents and the removal of polar low molecular weight components in equilibrium reactions [7].

## Background and theory

In general, membranes are very thin layers, which can have different structures. They are divided into porous membranes and solution-diffusion membranes. Porous membranes are well established in typical filtration processes e.g. micro-, ultra-, and nanofiltration. The particles to be separated have diameters between 10 and 1000 nanometers and will be held back from the membrane due to the fact that the pores of the membrane are smaller than the particle size.

If the size of the components to be separated is less than 1 nanometer – which is the case for many gaseous, vaporous and also liquid components that have to be removed from process streams – then mainly so called solution-diffusion membranes are used. This type of membrane does not have pores but free volume sites which exist due to restricted motion and packing density of the polymer chains.

**Figure 1:**
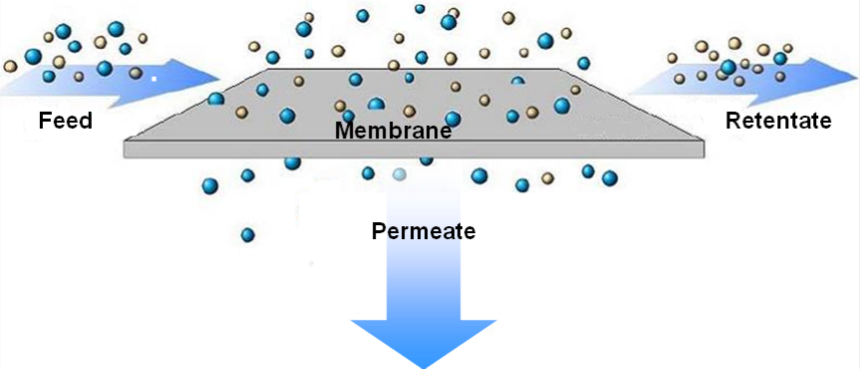
Membrane based separation process.

Figure 1 shows the principle of a membrane-based separation process. The feed mixture is transported along one side of the membrane and the different feed components permeate through the membrane at different rates. The stream leaving the membrane unit on the same side as the feed is depleted in the components which permeate preferentially. Consequently the stream, which is collected on the back side of the membrane, is enriched in the preferentially permeating component. The driving force for the mass transport through a polymeric membrane is the difference in the chemical potential between the feed and permeate side and depends on temperature, pressure and concentration. In pervaporation, a membrane based process for separating liquid mixtures and also employed in gas separation processes, the difference in chemical potential is mainly achieved by keeping the permeate pressure much lower than the feed pressure.

The mass transport through solution-diffusion membranes can be described with the solution-diffusion model [8]. Based on this model the components permeate through a polymeric membrane in a three step process, i.e., the sorption of the component on the membrane surface (feed side), the diffusion of the component through the free volume of the polymer and the desorption of the component on the permeate side of the membrane.

In order to optimize material properties for the solution-diffusion process through the polymer matrix, approaches can be made taking the molecular structure into account [9].

Thus, it is supposed that a certain free volume exists in polymers. This is the volume, which cannot be occupied by polymer chains due to conformational constraints. Within this free volume, transient gaps are formed which can accommodate, e.g., gas molecules. According to the driving force, the components have to be transported by successive movement between transient gaps close to the feed side to those close to the permeate side. The movement necessary for the transport of the components between the microvoids is possible due to thermal motion of segments of the polymer chains.

Polymeric membrane materials are generally characterized by the transport properties permeability and selectivity. Permeability is a measure of the productivity of the membrane and selectivity is a measure of the separation efficiency. For polymer films without any support, which are used as membrane materials in this review, the flux (*n_A_*), normalized by the transmembrane partial pressure (Δ*p_A_*) and thickness (ℓ), the permeability (*P_A_*) is defined, as shown in Equation 1.

[1]
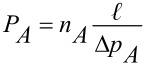


In gas separation devices the permeability values are typically reported in Barrer,

[2]
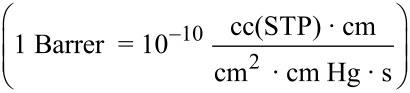


whereas in pervaporation processes the mass flux is reported in kg·μm·m^−2^·h^−1^. The ideal selectivity 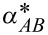 (i.e. pure feed components) between A and B is defined as the ratio of their permeabilities (Equation 3).

[3]
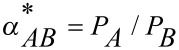


The permeability can be written as the product of the diffusion coefficient *D*, and the solubility coefficient *S* (Equation 4).

[4]
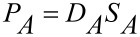


From this relationship, the ideal selectivity 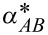 can be expressed by Equation 5.

[5]
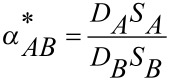


Thereby the solubility coefficient *S* is determined by the polymer-penetrant interactions and by the amount of free volume in the polymer. The average diffusion coefficient *D* is a measure of the mobility of the penetrants between the feed and permeate side of the membrane. The diffusion coefficient *D* depends on packing and motion of the polymer segments and on the size and shape of the penetrating molecules.

For binary feed mixtures in gas separation and also in pervaporation processes, the selectivity can be calculated from Equation 6.

[6]
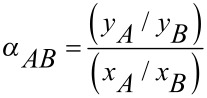


In which *x_i_* is the mole fraction of the preferred permeating component *i* on the feed side and *y_i_* is the mole fraction of the preferred permeating component *i* on the permeate side, as measured by gas chromatography.

## Results and Discussion

### Material selection

For the separation of liquid and gaseous mixtures, in general, porous as well as solution-diffusion membranes can be used. Although porous inorganic membranes, e.g., different zeolite types are characterized by their high thermal and chemical resistance, the application in large scale industrial processes seems to be rather difficult since preparing defect free membranes in huge areas is still difficult and expensive. The manufacturing of polymeric materials as composites or hollow fibers has been well established over the past 10–15 years, which is the reason why most of the commercial membrane units used for gas separation contain, e.g., polymer membranes [10]. Furthermore, strategies for new large-scale applications with polymeric membranes are under investigation [11–13].

Polymeric membrane materials can be divided into rubbery and glassy polymers. Extensive research in the area of gas separation has found correlations between the polymer structure and the separation characteristics. Thus glassy polymers show very attractive separation characteristics – high selectivity combined with medium permeability, whilst rubbery polymers show comparably low selectivity with high permeability for common gas pairs such as O_2_/N_2_, H_2_/CH_4_, CO_2_/CH_4_, etc. [14–16]. This correlation is demonstrated for the CO_2_/CH_4_ separation as shown in Table 1.

**Table 1:** Comparison of glassy and rubbery polymer membranes in CO_2_/CH_4_ separation [12].

Polymer	α* = P(CO_2_)/P(CH_4_)	P (CO_2_) [Barrer]

Cellulose derivatives	3	4550
Polycarbonates	11–33	75–15
Polyimides	15–25	110–6.5
Polydimethylsiloxane	55–65	23–0.6

**Figure 2:**
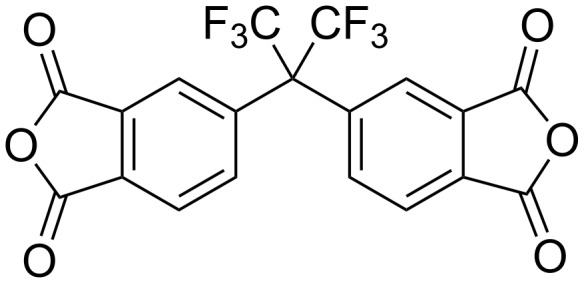
Chemical structure of the 6FDA (= 4,4′-hexafluoroisopropylidene diphthalic anhydride).

High selectivity is achieved with glassy polymers as a result of several factors, e.g., the lower free volume, a narrower distribution of the free volume as well as the lower flexibility of the polymer chains, compared to those of rubbery polymers. Within the class of glassy polymers, polyimides have been found to be very attractive as membrane materials because they have better separation characteristics compared to other glassy polymers, e.g., polycarbonates as also shown in Table 1. Additionally, polyimides offer good thermal and chemical resistance and are easy to process. Polyimide membranes are manufactured by several companies, e.g., Evonic, UBE, GKSS, MTR, etc., and are used mainly in gas separation processes but also find application in pervaporation and vapor recovery systems [17]. In order to improve the separation characteristics of this polymer class, systematic studies have been carried out over the last 10 years in order to find correlations between polyimide structure and separation performance. Thus it was found that with monomers having –CF_3_ groups, e.g. the 6FDA dianhydride, as shown in Figure 2, the bulky –CF_3_ groups restrict chain mobility and simultaneously chain packing, and consequently lead to significantly improved selectivity as well as permeability [18–22].

### Cross-linked polymers

**Figure 3:**
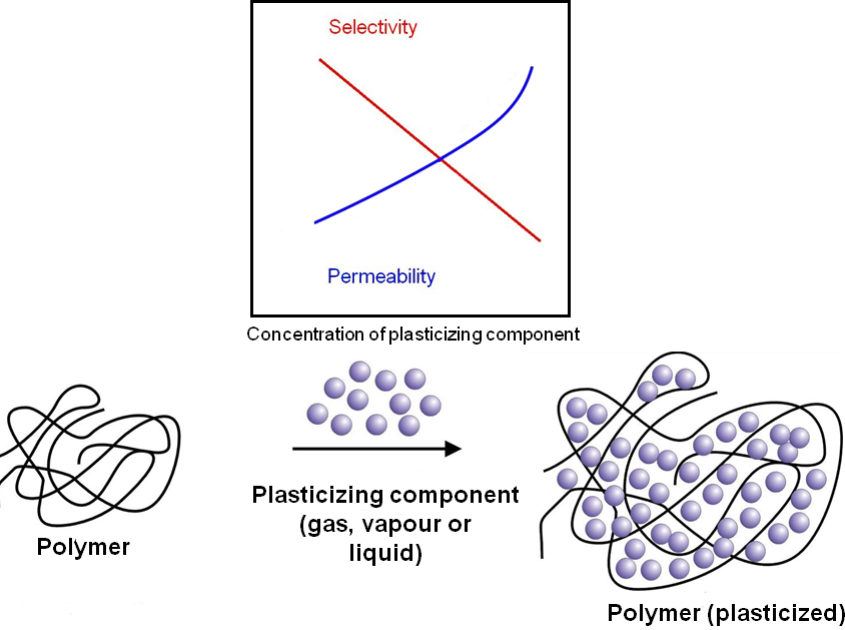
Plasticization phenomenon and resulting effects on separation characteristics.

6FDA-polyimides show excellent separation characteristics for different gaseous and liquid mixtures. However, in the presence of certain feed components plasticization occurs. Plasticization leads to an increase in the intermolecular distance and to a decrease in inter- and/or intra-molecular forces. As a consequence, the molecular motion of the polymer chains increases and as a result of this the permeabilities for all feed components also increase with an associated decrease in selectivity (Figure 3). It has been found in several studies that plasticization occurs if polyimides are exposed to high partial pressure of CO_2_ [23–24], hydrocarbons, e.g., propylene and propane [25–26] or ethylene oxide [27]. Strong plasticization can even lead to a partial dissolution of the membrane as it has been found in aromatic/aliphatic separation [28]. These results indicate that if new markets for membrane systems are found, then it will be definitely necessary to develop new plasticization resistant, robust membrane materials.

**Figure 4:**
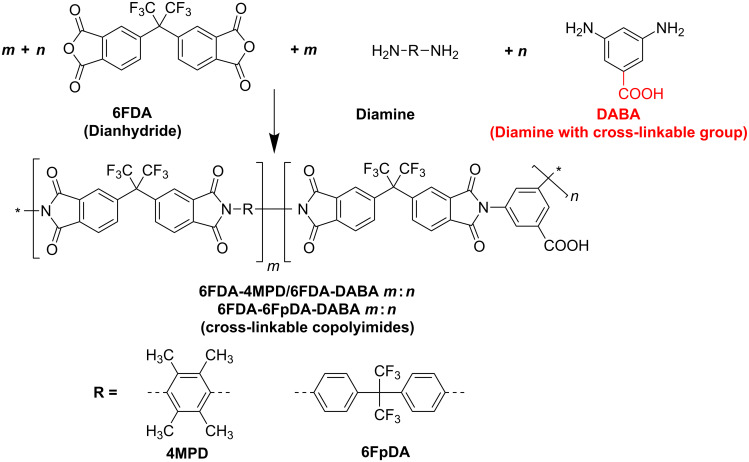
Synthesis of cross-linkable copolyimide structures.

Cross-linking of polymer structures has been found to be a suitable method to improve plasticization resistance as well as the separation characteristics for pervaporation [29–33] and gas separation [34–39]. Carboxy group containing 6FDA-based polyimides and copolyimides have been developed resulting in functional polymers which can be cross-linked or further modified. Consequently, plasticization effects can be reduced very efficiently [25,27,40–41]. As shown in Figure 4, such carboxy group containing copolyimides can be synthesized by using 3,5-diaminobenzoic acid as one of the monomers. Due to the lower reactivity of the carboxy groups on the diaminobenzoic acid (DABA) monomer compared to the dianhydride on the 6FDA, the carboxy groups are still present after the polymerization reaction and can be used in further reactions, e.g., cross-linking. Polymerization takes place in a two step reaction. Firstly, the purified diamino monomers were dissolved in dry *N*,*N*-dimethylacetamide and the 6FDA dianhydride is added under a nitrogen atmosphere in order to form the polyamic acid. After stirring for 24 h at room temperature, the viscous polyamic acid solution was chemically converted to the polyimide by treatment with a mixture of acetic anhydride and triethylamine. A detailed description of the synthesis is given in [28].

**Figure 5:**
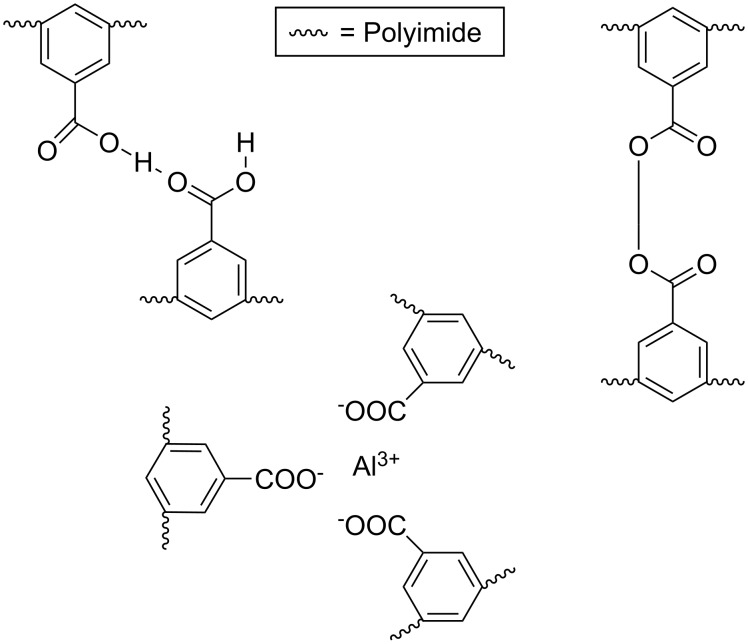
Investigated cross-linking variations (non cross-linked, covalently and ionically cross-linked).

In Figure 5 the three different types of copolyimides investigated are shown schematically. It is assumed that the polymer chains of copolyimides containing free carboxy groups are associated via hydrogen bonds. This is indicated by comparing the CO_2_ permeabilities for pure polyimides and copolyimides containing DABA. It was found that the presence of carboxy groups reduces the CO_2_ plasticization slightly due to the hydrogen bonds between the carboxylic acid groups [23]. Copolyimides with carboxy groups can be further modified via covalent cross-linking with, e.g., diols or diamines or cross-linked ionically with aluminium acetylacetonate or zirconium acetylacetonate as shown in Figure 5.

Covalent cross-linked membranes were prepared by dissolving the carboxy group containing copolyimide in dry *N*,*N*-dimethylacetamide and adding 6 times the stoichiometric amount of diol (based on the number of cross-linkable carboxy groups) together with *p*-toluenesulfonic acid as a catalyst to the casting solution. After the solvent has been evaporated at 70 °C, the membranes obtained are stored for another 24 h at 150 °C at 80 mbar in order to carry out the cross-linking reaction.

Ionically cross-linked membranes have been prepared by dissolving the carboxy group containing copolyimide in tetrahydrofuran (THF) and adding a stoichiometric amount of aluminium acetylacetonate or zircon(IV) acetylacetonate to the casting solution. After evaporation of the solvent, the membranes are dried in a vacuum oven at 150 °C and 80 mbar in order to perform the cross-linking reaction.

In the following sections it will be shown which cross-linking method, ionic or covalent, will be the most effective to reduce undesired plasticization effects occurring in different separations. Furthermore, different ionic cross-linkers are compared, since it was expected that higher cation loadings would lead to more effective cross-linking.

## Experimental set-up

According to Equation 4 and Equation 5, the separation characteristics, permeability and selectivity are dependent on the solubility and diffusivity of the single components in the membrane material. In order to estimate if the synthesized membrane polymer is suitable for a given separation problem, the solubility properties for the different feed components can be determined by means of gas or vapor sorption experiments. Diffusion coefficients can be determined by time dependent sorption measurements. For gas sorption experiments and for vapor sorption experiments, a microbalance and a quartz spring balance can be used, respectively [42–43]. It has been shown that polymeric membranes having significant differences in solubility for different feed components are particularly suitable for separating a mixture containing these components. However, sorption experiments do not give sufficient information about permeability and selectivity behavior if plasticization occurs. Therefore thorough gas permeation or pervaporation experiments are necessary with varying feed pressures and/or feed compositions. Only then reliable statements on potential applications of membrane systems are possible.

## Investigated separation problems

Several membrane-based separations are already well established commercially. However, the number of new potential applications is steadily increasing [5–6]. Therefore, it is absolutely necessary to develop new, economic and reliable membrane materials. In the following section, three examples of new applications, namely, the separation of gaseous olefin/paraffin mixtures, aromatic/aliphatic separation and the removal of carbon dioxide from natural gas a with high CO_2_ content, are discussed in detail.

## Olefin/paraffin separation

**Figure 6:**
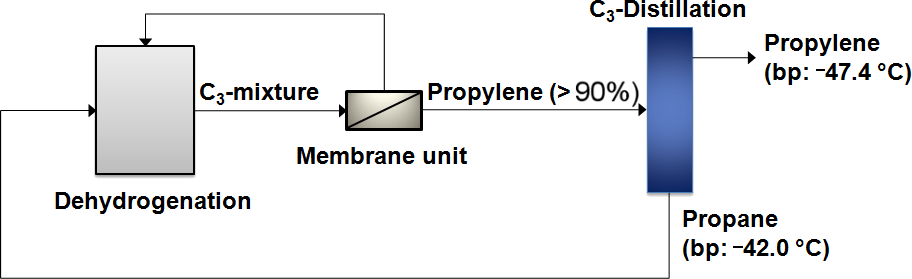
Hybrid process for the separation of propylene/propane.

The separation of olefin/paraffin mixtures is rather difficult because of the small differences in physical properties, e.g., boiling points. Currently, such separations are carried out by energy intensive low temperature distillation. Huge splitter columns are necessary to separate the mixtures of saturated and unsaturated hydrocarbons to obtain, e.g., propylene of sufficient purity for polymerization reactions. As shown in Figure 6, a hybrid process combining a membrane unit and a distillation column could lead, depending on the separation characteristics of the membrane material, to a significant reduction of the stream brought to the energy intensive splitter. Due to the fact that the separation train is more than half of the total cost of an olefin plant, a reduction of the splitter column is of high interest.

However, membrane based processes for olefin/paraffin separations are currently not possible because suitable membrane materials are not commercially available. It has been found that polymeric membranes, e.g., silicone rubber, polysulfone, cellulose acetate, PDMS, 1,2-polybutadiene and polyethylene are not suitable for this kind of separation because the separation factors are far too low [44–46]. Much better separation characteristics were achieved with polyimides as membrane materials [11,45,47–49].

**Figure 7:**
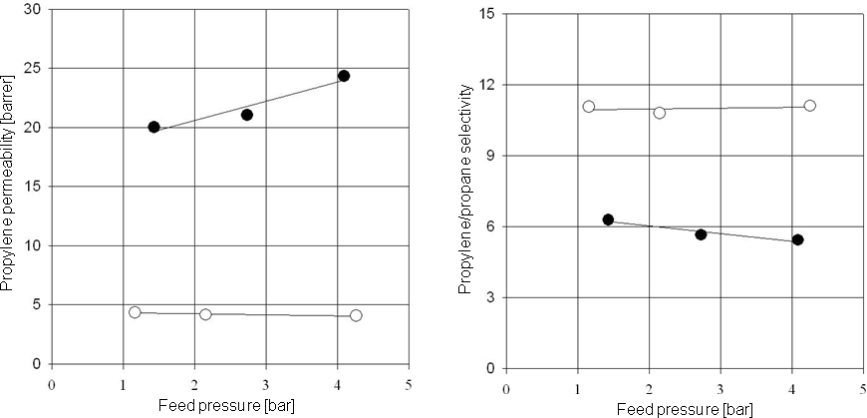
Total permeability (left) and selectivity (right) for the 6FDA-4MPD (●) and the 6FDA-4MPD/6FDA-DABA 4:1 copolyimide cross-linked with ethylene glycol (○) using a 50:50 propylene/propane feed mixture at 35 °C.

Unfortunately, polyimides are sensitive to plasticization due to higher hydrocarbons. It has been previously shown that neither ethane nor ethylene plasticize polyimide membranes but propane, propylene and higher hydrocarbon do [25,45,48–49]. In order to avoid undesirable plasticization effects, cross-linkable copolyimides have been synthesized, and their separation properties characterized. In Figure 7 the experimenstal data for the separation of a 50:50 propylene/propane mixture are shown. The temperature was 35 °C and the feed pressure was varied between 1 and 4.5 bar. In Figure 7, left-hand side, the total permeability is plotted versus the feed pressure, whilst on the right-hand side, the selectivity calculated from Equation 6 is shown as a function of the feed pressure.

As reference material, the 6FDA-4MPD was used as non cross-linkable membrane. In order to compare the propylene/propane separation characteristics of a 6FDA-4MPD polyimide membrane with a cross-linked copolyimide membrane, a copolyimide was synthesized substituting 20% of the 4MPD by the DABA diamine. The copolyimide obtained was the 6FDA-4MPD/6FDA-DABA 4:1. The structure is shown in Figure 4. Cross-linking of this type of polymer is possible due to the free carboxy groups and was performed by ethylene glycol treatment. As shown in Figure 7 for the reference polyimide 6FDA-4MPD, the permeability increases by 25% if the feed pressure is changed from 1.5 to 4 bar. Simultaneously, the selectivity for the propylene/propane separation decreases. This is caused by the increased mobility of the polymer chains during the swelling process as shown schematically in Figure 3. For the ethylene glycol cross-linked 6FDA-4MPD/6FDA-DABA 4:1 copolyimide, no plasticization effects were observed, since the mobility of the polymer chains is limited due to the cross-linking units. Therefore, the permeability as well as the selectivity remains constant. It should be also noted that a high selectivity of approximately 11 was found for the 6FDA-4MPD/6FDA-DABA 4:1 cross-linked with ethylene glycol, which means that with a 50:50 propylene/propane feed mixture a permeate concentration of more than 90% propylene can be achieved. This makes the membrane material very attractive for industrial applications.

## Aromatics/aliphatics separation

**Figure 8:**
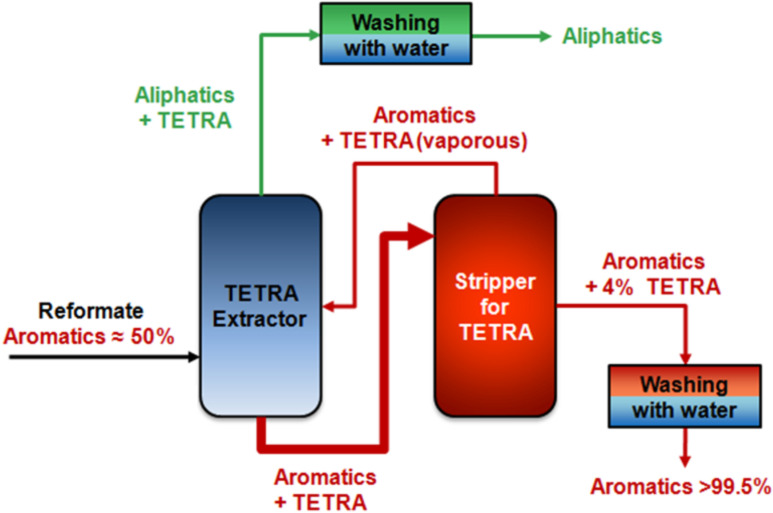
Conventional separation process for reformates containing extraction and stripping unit.

The separation of aromatics/aliphatics is receiving more and more attention, as the benzene content in gasoline is, by law in Europe, limited to less than 1%. Discussions on the reduction of toluene and polynuclear aromatic compounds in gasoline are ongoing. Figure 8 shows the conventional separation process for separating a reformate stream containing 40–50% aromatics in which the aromatics are mainly benzene, toluene with small amounts of xylenes. As shown in the process scheme, in the first step the extracting column separates the reformate stream into aliphatic and aromatic streams. The separation factor for the aromatics, using a TETRA (tetraethylene glycol)/water mixture as the extracting solvent, is between 2 and 3. The main disadvantage of the extracting unit is that a huge amount of TETRA/water is necessary, e.g., the ratio of aromatics/extracting solvent is 1:10. In the next process step a stripping unit is necessary, not only to separate the aromatics from TETRA but also to separate the aliphatics, which are still present in the extracting unit due to the low separation factor. The stream on top of the stripper column consists mainly of aliphatics but also contains aromatics as well as TETRA and therefore it has to be returned to the extraction column.

**Figure 9:**
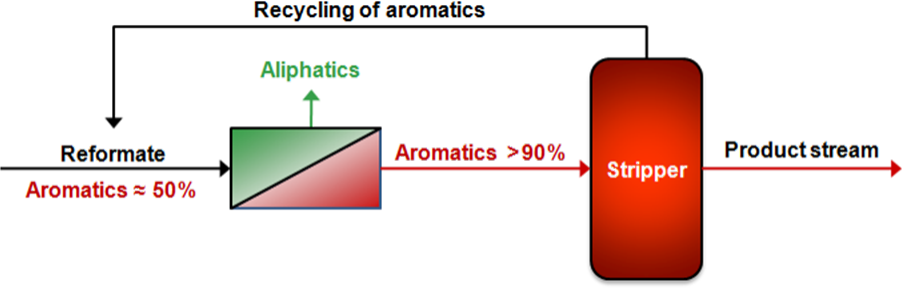
Hybrid process for the separation of aromatics/aliphatics.

Figure 9 shows how a membrane separation unit might be implemented into a conventional aromatic/aliphatic separation process. This design is advantageous because the complete extracting column is replaced by a single membrane unit. Therefore the process itself requires only minor changes, i.e. the splitting ratio of the streams coming from the splitter does not have to be changed. In the proposed hybrid process, the separation factors for the aromatics are twice as high using a membrane compared to the extractor running with TETRA. Furthermore, the membrane unit will drastically reduce the stream to the splitter as well as the stream coming out on top of the splitter.

**Figure 10:**
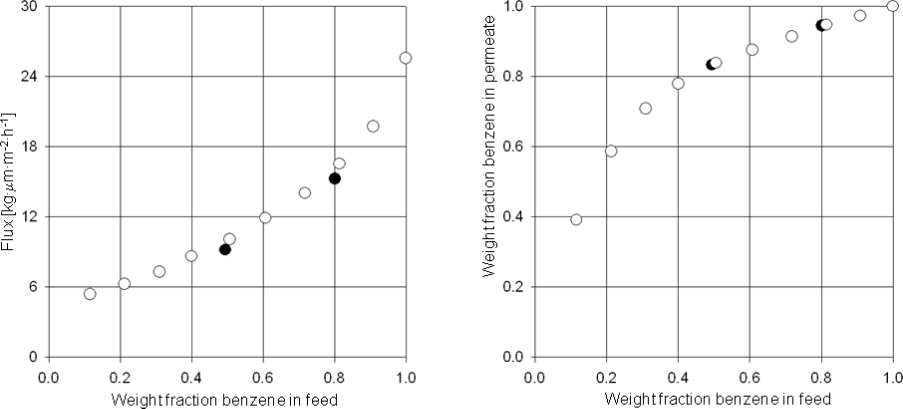
Pervaporation results for the 6FDA-6FpDA/6FDA-4MPD/6FDA-DABA 3:1:1 copolyimide cross-linked with ethylene glycol (first run (●) and second run (○) using benzene/cyclohexane mixtures at 60 °C and a permeate pressure of 10–20 mbar (data taken from [28]).

Although the feed concentration for the aromatics in reformate streams is usually between 40% and 50%, it sometimes happens that much higher concentrations occur over a short time period. Therefore it is important to ensure that the membrane material is stable and additionally, that the separation characteristics – especially the selectivity – will not change drastically. It has been demonstrated that polyimides in principle are suitable for the separation of aromatic/aliphatic mixtures but unless they are cross-linked, the stability is poor especially at high aromatic concentrations and elevated feed temperatures. In order to circumvent these problems in practical applications, different copolyimides have been synthesized and their stabilities after exposure to high benzene concentrations tested. Figure 10 shows the 6FDA-6FpDA/6FDA-4MPD/6FDA-DABA 3:1:1 cross-linked with ethylene glycol in a stability experiment. This experiment was started by running pervaporation experiments, with benzene/cyclohexane mixtures with benzene concentrations of 50 and 80 wt %. After the membrane was exposed to 80% benzene, the feed mixture was exchanged and then the separation characteristics for benzene concentrations of 10 wt % up to 100 wt % in the feed were investigated. In this experiment it can be clearly seen that covalently cross-linked copolyimides are very stable towards high benzene concentrations [28]. No change in selectivity was observed after the cross-linked membrane was exposed to high aromatic concentrations in the feed. It has been found that cross-linked copolyimide membranes can even be exposed to 100% aromatics before starting separation experiments without any loss in selectivity. In certain cases even increased permeability without any loss in selectivity has been observed.

**Figure 11:**
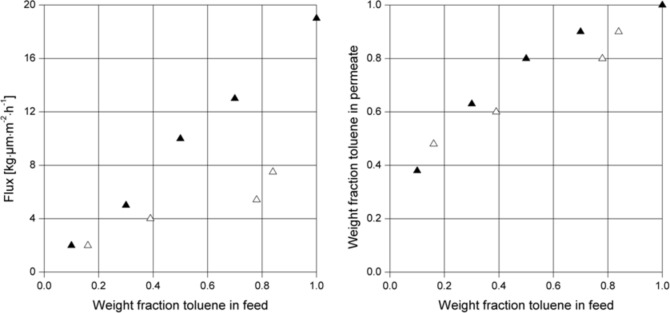
Pervaporation results for 6FDA-4MPD/6FDA-DABA 4:1 copolyimide (non cross-linked) conditioned in pure toluene (▲) and in a mixture of 90:10 toluene/cyclohexane (Δ). After conditioning, the measurements were performed using toluene/cyclohexane mixtures at 60 °C and a permeate pressure of 20–25 mbar.

Pre-treatment of the membrane material prior to its use is referred to as conditioning. The effects of varying the composition of the conditioning agent were also investigated in this work. 6FDA-4MPD/6FDA-DABA 4:1 was used as the basic membrane material and non cross-linked membranes were prepared and conditioned in pure toluene and in a mixture of 90:10 toluene/cyclohexane, respectively. The results of the pervaporation experiments at 60 °C and at a permeate pressure of 20–25 mbar are shown in Figure 11.

It can be seen clearly that the membrane conditioned in pure toluene shows a much higher flux than the one conditioned in 90:10 toluene/cyclohexane. This effect is even more pronounced at higher toluene concentrations in feed. Remarkably, there is no significant difference in the selectivity for the different conditioned membranes within experimental error. From our experience in pervaporation experiments, the error range for the selectivity is ±10%, whereas for the flux it could be as much as 15%, depending on the homogeneity of the membranes. However, from the experiments performed, it can be concluded, that with appropriate conditioning, fluxes can be increased without any loss in selectivity.

Further experiments have been carried out in order to investigate the separation characteristics for covalently cross-linked copolyimide membranes compared to ionically cross-linked copolyimide membranes and non cross-linked membranes prepared from the same type of polymer. The polymer 6FDA-4MPD/6FDA-DABA 4:1 copolyimide was used and the covalent cross-linker was 1,4-butanediol. An ionically cross-linked membrane was prepared from the basic polymer material by adding 10% of the stoichiometric amount of cross-linker (based on the free carboxy groups of the polymer). For the ionic cross-linking, zircon(IV) acetylacetonate was used since it was expected to be more efficient than aluminium acetylacetonate because of the higher charge of the cation formed during the cross-linking reaction. The mixture to be separated consisted of toluene and cyclohexane and the experiments were carried out at 60 °C at a permeate pressure of 20–25 mbar.

**Figure 12:**
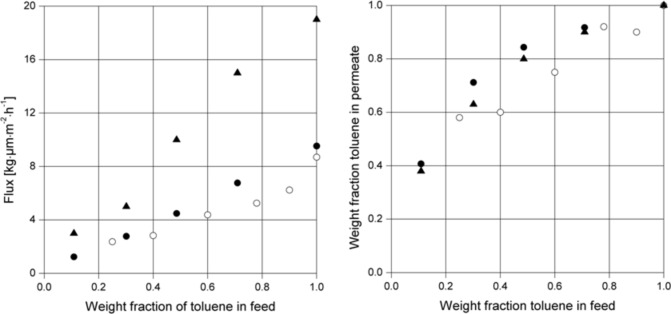
Pervaporation results for conditioned 6FDA-4MPD/6FDA-DABA 4:1 copolyimide membranes, 100% cross-linked with 1,4-butanediol (●) and 10% cross-linked with zircon(IV) acetylacetonate (○) and non cross-linked (▲) using a toluene/cyclohexane mixture at 60 °C. Permeate pressure was kept between 20 and 25 mbar.

The results obtained (Figure 12) show that the flux of a non cross-linked 6FDA-4MPD/6FDA-DABA 4:1 membrane is much higher than the fluxes of the covalently and the ionically cross-linked membranes. In addition, it can be seen that the separation properties of the ionically cross-linked 6FDA-4MPD/6FDA-DABA 4:1 membrane is significantly lower, especially at higher aromatic feed concentrations compared to the covalently cross-linked membrane and the non cross-linked membrane.

From the experiments it can be concluded that conditioning of the membrane material is favourable since the flux can be increased without a loss in selectivity. It can be also seen that cross-linking is not necessary for the aromatic/aliphatic separation at moderate temperatures, e.g. 60 °C, but it presumably will increase the life time of the membrane with respect to the proposed hybrid process shown in Figure 9. It is obvious that if a 50:50 aromatic/aliphatic mixture is treated with a membrane unit (as shown in Figure 9), this could lead according to the results presented in Figure 12 to a 90% aromatics containing stream on the back side of the membrane. As a result the stream to the extraction column can be reduced by half, with a reduction of both extracting solvents and energy.

## Natural gas treatment

**Figure 13:**
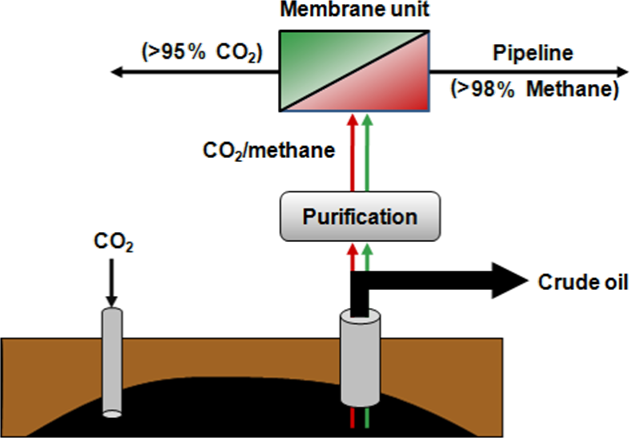
Hybrid process for the removal of CO_2_ in tertiary oil production processes.

In the area of natural gas treatment a number of different applications are of great interest. In tertiary oil production supercritical CO_2_ is introduced into the oil field, especially if the oil is distributed in porous layers. As shown in Figure 13, the stream coming out of the oil field then contains the crude oil as well as gaseous compounds e.g. natural gas, higher hydrocarbons, H_2_S and a small amount of water. However, it is important to hold back the CO_2_ present in this stream because it is well known that CO_2_ is one of various compounds responsible for the greenhouse effect. Figure 13 shows how a membrane unit can be implemented in such a process, so that the CO_2_ is removed through the membrane and can then be re-used after compression. With this process not only could the emission of CO_2_ be drastically reduced but also the natural gas can be recovered in this case instead of burning it which is generally carried out if the quantity and quality are too low.

**Figure 14:**
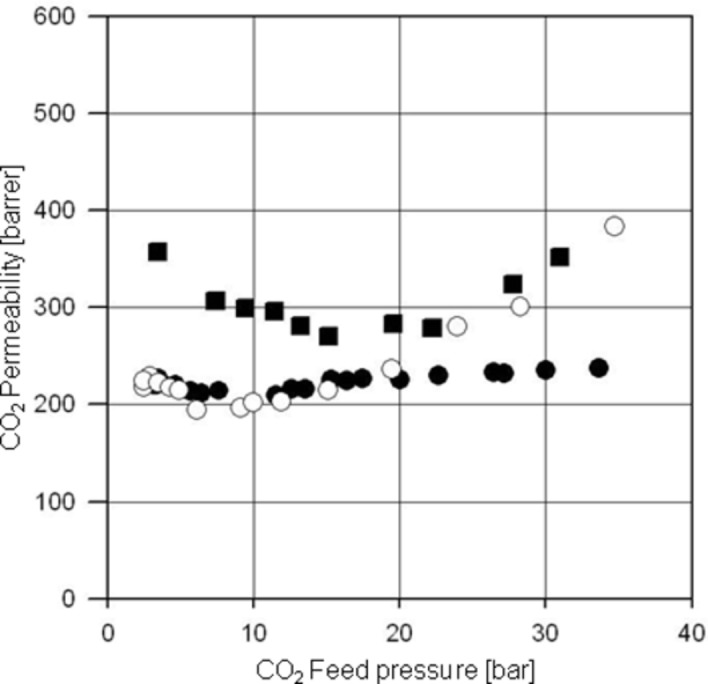
Pure CO_2_ permeabilities at 35 °C for the 6FDA-4MPD (■), the 6FDA-4MPD/6FDA-DABA 4:1 copolyimide ionically cross-linked with aluminium acetylacetonate (○) and covalently cross-linked with ethylene glycol (●).

Another very interesting application is the treatment of natural gas in offshore deposits. So far a huge number of gas resources are known worldwide, which cannot be exploited because of the high CO_2_ content and the high pressure of the mixture. For economic reasons more and more membrane based processes in natural gas treatments are operated with polyimides as the membrane material instead of cellulose derivatives, since the intrinsic transport properties for the polyimides are much better. However, strong plasticization effects occur with non cross-linked polyimides generally at 10–20 bar partial CO_2_ pressure in the feed. Therefore, in this context cross-linkable copolyimides are of interest because they offer plasticization resistance up to much higher CO_2_ pressures and, in addition, they have better chemical resistance. In Figure 14 the higher plasticization resistance of cross-linked copolyimides compared to non cross-linked polyimides is illustrated. Plasticization resistance for different cross-linked copolyimides (covalently versus ionically) has been investigated in order to ascertain the most suitable cross-linking technique for this particular application.

It can be seen that the non cross-linkable reference polyimide, 6FDA-4MPD indeed starts plasticizing at a pure CO_2_ pressure of approximately 15 bar. The plasticization is indicated by a large increase in permeability caused by increasing segmental motion of the polymer chains. Figure 14 shows that the ionically cross-linked material also plasticizes at very low CO_2_ pressures whereas the covalently cross-linked membrane is resistant to plasticization up to a CO_2_ pressure of approximately 30 bar. Similar effects have been found for different copolyimide structures [40].

It is assumed that ionic cross-linking leads to a much lower plasticization resistance compared to covalent cross-linking because ionic aggregates are formed due to electrostatic interactions, in this case between aluminium cations and carboxylate anions. Thus, heterogeneous regions with ionic and non-ionic domains are produced. The non-ionic regions consist mainly of polymer chains which are sensitive to plasticization in the presence of strong plasticization agents such as CO_2_. Another plausible reason might be that the CO_2_ with its strong solvation effect is able to weaken the ionic interactions in the ionic regions.

In Table 2 pure CO_2_ permeabilities and ideal CO_2_/CH_4_ selectivities, which are the ratio of pure gas permeabilities, are reported for experiments performed at 10 bar feed pressure and 35 °C. Compared to the reference polyimide 6FDA-4MPD, the modified but non cross-linked 6FDA-4MPD/6FDA-DABA 4:1 copolyimide shows a much lower permeability but higher selectivity. The lower permeability results because the DABA in the polymer structure provides a much lower free volume than the 4MPD. With both cross-linked versions of the 6FDA-4MPD/6FDA-DABA 4:1, the permeability can be increased whereas the selectivity is not changed significantly compared to the non cross-linked 4:1 copolyimide. However, the ideal selectivities obtained with the different cross-linked 6FDA-4MPD/6FDA-DABA 4:1 copolyimides are not high enough to be commercially attractive. It should be noted that these are ideal selectivities and often the selectivities obtained with feed mixtures are even lower due to the fact that the diffusivity of the individual components are influenced by each other. This leads to effects where the slower permeating component is accelerated whereas the faster permeating component is slowed down.

**Table 2:** CO_2_ Permeability for different copolyimides at 35 °C and 10 bar feed pressure.

Polymer	P(CO_2_) [Barrer]	α* = P(CO_2_)/P(CH_4_)

6FDA-4MPD	300.0	15.6
6FDA-4MPD/6FDA-DABA 4:1	129.3	23
6FDA-4MPD/6FDA-DABA 4:1(covalently cross-linked)	221.1	23
6FDA-4MPD/6FDA-DABA 4:1 (ionically cross-linked)	200.5	20

In order to increase the selectivity the basic polymer structure was slightly modified. Thus, part of the 4MPD was substituted by the 6FpDA which is well known for both its high selectivity and high permeability due to the –CF_3_ groups, which being bulky groups cause restricted rotation around the main polymer chain and also provide a high free volume.

**Figure 15:**
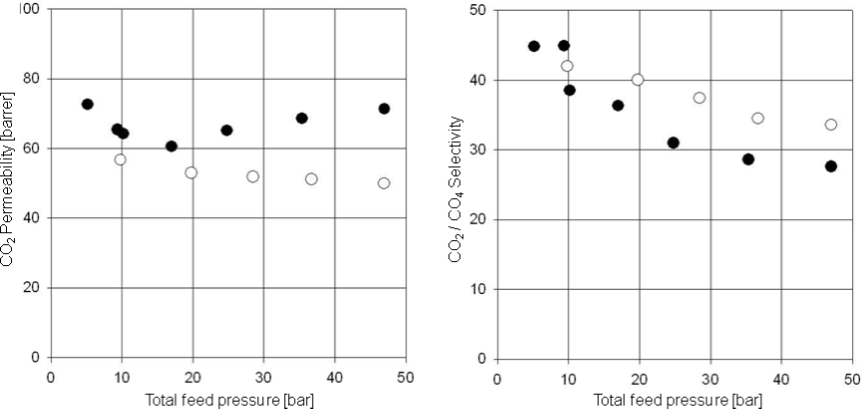
CO_2_/CH_4_ separation characteristics for the 6FDA-4MPD/6FDA-DABA 4:1 copolyimide ionically cross-linked with aluminium acetylacetonate (●) and covalently cross-linked with ethylene glycol (○) at 35 °C using a 50:50 CO_2_/CH_4_ feed gas mixture.

In Figure 15 the mixed gas results obtained with a CO_2_/CH_4_ feed mixture at 35 °C are presented for the 6FDA-6FpDA/6FDA-4MPD/6FDA-DABA 3:1:1 cross-linked covalently with ethylene glycol and the ionically cross-linked with aluminium acetylacetonate. The results mirrored those found for the 4:1 copolyimide. The ionically cross-linked structure began to plasticize very early (approximately at a CO_2_ partial pressure of 20 to 25 bar), whereas the ethylene glycol cross-linked structure shows nearly constant permeability and a selectivity which is approximately 20% higher than the selectivity achieved with the ionically cross-linked structure. From the separation diagram it is also obvious that the selectivity is dependent on the feed pressure. With increasing feed pressure the usual decrease in selectivity was found, which is due to the interactions between the single components in the feed mixture as previously discussed. However, the modified polymer structure shows good selectivity for CO_2_ over methane between 30 and 40, which indeed is attractive for commercial applications because it is much higher than the separation factors of the cellulose derivatives in current use.

## Conclusion

It has been shown for the separation of high-pressure mixtures of CO_2_/CH_4_ as well as propylene/propane mixtures that the prepared cross-linked copolyimide membranes are much more plasticization resistant than non cross-linkable polyimide membranes used as reference substances. In the separation of aromatic/aliphatic mixtures, it was found that with conditioning, high fluxes combined with high selectivities can be achieved, e.g., with a 50:50 toluene/cyclohexane mixture a toluene concentration in permeate of approximately 85% can be reached. In the propylene/propane separation a 50:50 feed mixture could be concentrated to more than 90% propylene in permeate with a covalently cross-linked copolyimide structure of 6FDA-4MPD/6FDA-DABA 4:1. It was found that in the pressure range investigated (up to 4.2 bar) no plasticization was found with the cross-linked structures, whereas for the non cross-linked structure plasticization starts at a very low feed pressure of around 2 bar.

For the removal of CO_2_ from natural gas, experiments with different cross-linked membranes showed that the covalently cross-linked copolyimide structure shows a much higher plasticization resistance and better selectivity in mixed gas experiments compared to the ionically cross-linked structures.

## References

[1] Ullman’s Encyclopedia of Industrial Chemistry (2002). C. Judson King University of California.

[2] Davis J C, Valus R J, Eshragi R, Velikoff A E (1993). Sep Sci Technol.

[3] Strathmann H (2001). AIChE J.

[4] Ohlrogge K, Stürken K, Nunez S P, Peinemann K-V (2001). The separation of organic vapors from gas streams by means of membrane technology, Membrane technology in the chemical industry.

[5] Baker R W (2002). Ind Eng Chem Res.

[6] Available from: http://www.borsig-china.com/#productrecovery

[7] Available from: http://www.sulzerchemtech.com

[8] Graham T (1867). Phil Trans/Ann Chem Pharm.

[9] Xiao Y, Low B T, Hosseini S S, Chung T S, Paul D R (2009). Prog Polym Sci.

[10] Maier G (1998). Angew Chem, Int Ed.

[11] Koros W J, Mahajan R (2000). J Membr Sci.

[12] Lin L, Kong Y, Yang J, Shi D, Xie K, Zhang Y (2007). J Membr Sci.

[13] Lipnizki F, Field R W, Ten P K (1999). J Membr Sci.

[14] Robeson L M (1991). J Membr Sci.

[15] Stern S A (1994). J Membr Sci.

[16] Robeson L M (2008). J Membr Sci.

[17] Ohya H, Kudryavtsev V V, Semenova S I (1996). Polyimide Membranes – Applications, Fabrications and Properties.

[18] Tanaka K, Okano M, Kita H, Okamoto K-i, Nishi S (1994). Polym J.

[19] Coleman M R, Koros W J (1990). J Membr Sci.

[20] Mikawa M, Nagaoka S, Kawakami H (1999). J Membr Sci.

[21] Lin W-H, Chung T-S (2001). J Membr Sci.

[22] Tanaka K, Kita H, Okano M, Okamoto K-i (1992). Polymer.

[23] Staudt-Bickel C, Koros W J (1999). J Membr Sci.

[24] Wessling M, Schoeman S, van der Boomgaard T, Smolders C A (1991). Gas Sep Purif.

[25] Staudt-Bickel C, Koros W J (2000). J Membr Sci.

[26] Kim J H, Koros W J, Paul D R (2006). J Membr Sci.

[27] Schiewe B, Staudt-Bickel C, Vuin A, Wegner G (2001). ChemPhysChem.

[28] Jizhong R, Staudt-Bickel C, Lichtenthaler R N (2001). Sep Purif Technol.

[29] Inui K, Noguchi T, Miyata T, Uragami T (1999). J Appl Polym Sci.

[30] Cao B, Hinode H, Kajiuchi T (1999). J Membr Sci.

[31] Fang J, Tanaka K, Kita H, Okamoto K (1999). Polymer.

[32] (1997). AICHE Annual Meeting.

[33] Matsui S, Paul D R (2002). J Membr Sci.

[34] Rezac M E, Sorensen E T, Beckman H W (1997). J Membr Sci.

[35] Rezac M E, Schoberl B (1999). J Membr Sci.

[36] Bos A, Punt I G, Wessling M, Strathmann H (1998). J Polym Sci, Polym Phys Ed.

[37] McCaig M S, Paul D R (1999). Polymer.

[38] Wright C T, Paul D R (1997). J Membr Sci.

[39] Kita H, Inada T, Tanaka K, Okamoto K-i (1994). J Membr Sci.

[40] Wind J D, Staudt-Bickel C, Koros W J, Paul D R (2002). Ind Eng Chem Res.

[41] Pithan F, Staudt-Bickel C, Heß S, Lichtenthaler R N (2002). ChemPhysChem.

[42] Funke H (1991). Sorption von Reingasen in Polymeren und an Zeolithen bei Drücken.

[43] Cen Y, Staudt-Bickel C, Lichtenthaler R N (2002). J Membr Sci.

[44] Ito A, Hwang S-T (1989). J Appl Polym Sci.

[45] Tanaka K, Taguchi A, Hao J, Kita H, Okamoto K (1996). J Membr Sci.

[46] Shimazu A, Ikeda K, Hachisuka H (1998). Method of selectively separating unsaturated hydrocarbon.. U.S. Patent 5.

[47] Lee K R, Hwang S-T (1992). J Membr Sci.

[48] Krol J J, Boerrigter M, Koops G H (2001). J Membr Sci.

[49] Shimazu A, Miyazaki T, Maeda M, Ikeda K (2000). J Polym Sci, Part B: Polym Phys.

